# On the Use of Monesia

**Published:** 1840-08-15

**Authors:** Robert Burns

**Affiliations:** Frankford, Philadelphia Co.


// MEDICAL EXAMINER.

DEVOTED TO MEDICINE, SURGERY, AND THE COLLATERAL SCIENCES.

No. 33.] PHILADELPHIA, SATURDAY, AUGUST 15, 1840. [Vol. III.

### ON THE USE OF MONESIA.

BY ROBERT BURNS, M. D.

To the Editors of the Medical Examiner.

*Gentlemen,*—Being much interested with the account given of monesia, in the third volume of the Examiner, (page 207, also at page 215,) I resolved on testing for myself the powers of this new therapeutic agent, when suitable occasions presented; accordingly, agreeable to reference, I called at the pharmaceutical establishment of Mr. F. Brown, Chestnut street, Philadelphia, on the 28th of April last, and through his politeness procured ʒi. of the extract. At this time I had under my care a severe case of menorrhagia, in which I was desirous to try the monesia, but on my return from the city on the day above mentioned, I found my patient improving under the use of the Acetas Plumbi, injections of vinegar and water, and cold applications to the hypogastrium; which treatment was continued with ultimate success, without having recourse to the monesia. Since then, I have used it in two cases of menorrhagia, one of hæmoptysis, and two of chronic diarrhœa; the latter supervening in infants from severe attacks of cholera infantum,—brief notices of which cases I copy from my note book, and should you deem it proper to give them publicity, I hope other remarks will be elicited upon this highly useful and safe medicine, from those who are more capable of doing justice to the subject than I am.

Case I.—Mrs. S——l, a lady of nervo-sanguineous temperament, was seized with a severe attack of menorrhagia, on the 7th of May last. On my arrival she was found considerably exhausted, with cold skin and feeble pulse; being provided with the monesia, I administered it according to the following form:

℞. Ext. Monesiæ, ʒss., ft. pulv. et div. in ch. x.

One of these powders was given immediately, in sugar and water, and directions left to give one every hour until the discharge subsided, in conjunction with which the usual cold applications were ordered to be used—with perfect rest, quietude, and cold drinks.

*Friday, May* 8th. Patient much better, the discharge having ceased after the administration of the second powder; on taking which, she experienced slight pain in the head, but which was of short duration. No other treatment was necessary in this case, with the exception of ℥i. Ol. Ricini on the following day.

Case II.—Mr. J. B——s, a young man of sanguineous temperament, subject for several years to periodical hæmoptysis, was, in the month of May, seized with an attack of unusual severity, and of a very alarming aspect. The monesia was given very frequently in three grain doses as above, being guided in the frequency of its administration more by the discharge of blood, than by a fixed period of time. In the course of two hours, he took from between ʒss. to ℈ii.; each dose was accompanied with a cessation of the discharge for the time being; but no sooner had the monesia spent its power, (if I may be allowed the phrase,) than it recurred, owing, as I felt fully persuaded, to too great action of the heart and arteries, and from the sanguineous congestion which existed in the system. A further dependence could no longer be placed on the monesia until this state of things was removed. Guided by these pathological views, I abstracted blood from the arm, until a decided impression was made on the pulse, and symptoms of approaching syncope induced, which required, for this purpose, about twenty ounces. The hæmoptysis did not entirely cease after venesection; accordingly, recourse was again had to the monesia, which now proved beneficial, and my patient was soon convalescent; since which time, he has had no return of the disease, and is in the enjoyment of good health.

Case III.—Mrs. O.'s child, between eight and nine months old, had an attack of cholera infantum, in July last, for which the usual remedies were employed with entire success, and convalescence advanced rapidly; in consequence, however, of imprudence in diet, a diarrhœa supervened, by which the child was becoming much debilitated. Recourse was had to calomel and Pulv. Opii et Ipecac. the warm bath, emollient cataplasms to the abdomen, and various astringent and cretaceous preparations, with partial benefit; still the disease was not subdued. On the 9th of August the monesia was prescribed as follows:

℞. Pulv. Ext. Monesiæ, gr. vi., Aquæ Font. f℥iss., M. ft. Solut. S. A tea-spoonful to be given every two hours.

*Aug.* 10. Since yesterday, five doses of the monesia, containing two and a half grains of the extract, were given, with complete success. Ordered the solution to be discontinued, and given only as occasion might require. This child has continued to do well up to the present time.

Case IV.—L. F——, a child eight months old, of feeble phlegmatic constitution, having lost its mother soon after birth, it was under the necessity of being brought up artificially; through, however, the assiduity and kindness of its nurse, it became a healthy and thriving child. By an unfortunate change of nurse, and error in the preparation of its food, it soon became the subject of cholera infantum, which, as in the preceding case, yielded to small doses of calomel and Dover's powder, in conjunction with the warm bath, &c. Afterwards, an exhausting diarrhœa came on, for which various preparations were used, without much advantage, and the disease seemed likely to terminate the existence of the little sufferer. At this time, the dejections occurred as often as twenty times a-day. On the 12th of August, I commenced the monesia according to the formula above mentioned, with directions to give a teaspoonful every two hours. In two days, by the use of this valuable medicine, the discharges were reduced to three or four a-day, improved both in colour and consistency.

*Aug.* 17. Dejections more frequent, amounting to five or six a-day, for the last two days; child very fretful—more, however, from a change of nurse, than disease. Ordered the monesia to be given, with mucilage of G. Acaciæ.

18th. Much better; has had only three discharges from the bowels since yesterday; aphthæ on tongue and inside of lips, with much salivary discharge; for which the following powder was prescribed:

℞. Bi-Borat. Sodæ, Sacch. Alb., āā ʒii.; Carb. Ferri Præp., ℈ss., M. ft. pulv.

About five grains of this powder was directed to be placed in the mouth three or four times daily. The monesia ordered to be continued *pro re nata.*

21st. Aphthæ have entirely disappeared, and the child, upon the whole, much improved, which improvement continues up to the present time.

Case V.—Catharine H——, a coloured woman, subject to frequent attacks of menorrhagia, some of which have continued, as I was informed, for weeks together, was again taken very ill on Sunday, Aug. 16, which she attributed to wet and fatigue. When I arrived, she was prostrated, and unable to raise her head from the pillow without feeling giddy and faint; she had considerable warmth of skin; slow but tolerably firm pulse. Prescribed the following:

℞. Pulv. Ext. Monesiæ, gr. xii., div. in ch. no. iv. Sig. One every two hours until the discharge ceased; in addition, cold applications were suggested.

*Monday, Aug.* 17th. Patient much better; skin cool; pulse softer than yesterday; discharge ceased after taking the second powder, which produced pain in the left side, "running down," as she termed it, to the uterus: this, however, soon passed away. Desired her to continue a-bed, and use the powders as directed, should occasion require.

18th. She is to-day so well as to sit up, and do needle-work; discharge has nearly gone altogether. On making some inquiry relative to the comparative effects of the monesia and the medicines she had taken on former occasions, which, from her description, I presumed to be the Acetas Plumbi, principally, she said she never took medicine so powerful as that I had given her: neither did she obtain relief so quick on any previous occasion. She repeatedly said "it took hold of the disease right away," and "went right to the part"—meaning, I apprehend, its producing uterine contraction. She continues to do well.

From the experience I have had, as above stated, and the testimony of others who have employed the monesia, I consider it a valuable acquisition to the materia medica. In diseases of an active character, where inflammation or plethora exists, I am inclined to believe it is inadmissable; but this state of the system being removed, or the disease being of a chronic character, I esteem it a medicine of no ordinary utility. In infantile diarrhœas, I consider it peculiarly adapted, inasmuch as with children there is generally great difficulty in the administration of medicines, owing to the disagreeable taste of most of them. With monesia this does not obtain, it being either in substance or aqueous solution, rather agreeable to the taste than otherwise. In menorrhagia, it really seems to act with much promptitude; surpassing, according to my opportunities of observation, the Acetas Plumbi, and resembling closely the action of the Secale Cornutum in producing uterine contraction.

Fearing lest I should exhaust your patience by further extending these remarks, I leave the subject for the present, hoping at a future period to send you the results of a more extended experience.

*Frankford, Philadelphia Co., August* 28, 1840.

